# Complete genome sequence of *Streptosporangium roseum* type strain (NI 9100^T^)

**DOI:** 10.4056/sigs.631049

**Published:** 2010-01-28

**Authors:** Matt Nolan, Johannes Sikorski, Marlen Jando, Susan Lucas, Alla Lapidus, Tijana Glavina Del Rio, Feng Chen, Hope Tice, Sam Pitluck, Jan-Fang Cheng, Olga Chertkov, David Sims, Linda Meincke, Thomas Brettin, Cliff Han, John C. Detter, David Bruce, Lynne Goodwin, Miriam Land, Loren Hauser, Yun-Juan Chang, Cynthia D. Jeffries, Natalia Ivanova, Konstantinos Mavromatis, Natalia Mikhailova, Amy Chen, Krishna Palaniappan, Patrick Chain, Manfred Rohde, Markus Göker, Jim Bristow, Jonathan A. Eisen, Victor Markowitz, Philip Hugenholtz, Nikos C. Kyrpides, Hans-Peter Klenk

**Affiliations:** 1 DOE Joint Genome Institute, Walnut Creek, California, USA; 2 DSMZ - German Collection of Microorganisms and Cell Cultures GmbH, Braunschweig, Germany; 3 Los Alamos National Laboratory, Bioscience Division, Los Alamos, New Mexico, USA; 4 Oak Ridge National Laboratory, Oak Ridge, Tennessee, USA; 5 Biological Data Management and Technology Center, Lawrence Berkeley National Laboratory, Berkeley, California, USA; 6 HZI – Helmholtz Centre for Infection Research, Braunschweig, Germany; 7 University of California Davis Genome Center, Davis, California, USA

**Keywords:** *Sporangia*, vegetative and aerial mycelia, aerobic, non-motile, non-motile spores, Gram-positive, *Streptosporangiaceae*, *S. cloviforme*

## Abstract

*Streptosporangium roseum* Crauch 1955 is the type strain of the species which is the type species of the genus *Streptosporangium*. The ‘pinkish coiled *Streptomyces*-like organism with a spore case’ was isolated from vegetable garden soil in 1955. Here we describe the features of this organism, together with the complete genome sequence and annotation. This is the first completed genome sequence of a member of the family *Streptosporangiaceae*, and the second largest microbial genome sequence ever deciphered. The 10,369,518 bp long genome with its 9421 protein-coding and 80 RNA genes is a part of the *** G****enomic* *** E****ncyclopedia of* *** B****acteria and* *** A****rchaea * project.

## Introduction

Strain NI 9100^T^ (= DSM 43021 = ATCC 12428 = JCM 3005) is the type strain of the species *Streptosporangium roseum*, which is the type species of the genus *Streptosporangium*, the type genus of the actinobacterial suborder *Streptosporanineae* [1-4]. S. roseum NI 9100^T^ was isolated from vegetable garden soil and first described by Crouch in 1955 [2,4]. The name derives from ‘strepto’ from Greek meaning ‘coiled’ combined with ‘sporangium’, Latin for ‘spore case’, to mean ‘streptomyces-like’ but with sporangia [2,4]. The species epithet ‘roseum’ derives from the pinkish color on potato dextrose agar [2]. Here we present a summary classification and a set of features for *S. roseum* NI 9100^T^, together with the description of the complete genomic sequencing and annotation.

## Classification and features

The 16S rRNA genes of the thirteen other validly named species currently ascribed to the genus *Streptosporangium* share 96-100% (S. vulgare [5]) sequence identity with NI 9100^T^, but *S. claviforme* (94%) [6,7] apparently does not belong to this genus (but to the genus *Herbidospora*) and thus has been excluded from phylogenetic analysis (see below). Two reference strains, DSM 43871 (X89949), and DSM 44111 (X89947), differ by just one nucleotide from strain NI 9100^T^, whereas the effectively published named species ‘*S. koreanum*’ DSM 44110 [99.9%, 5], ‘*S. brasiliense*’ DSM 44109 [99.4%, 5] and ‘*S. rubrum*’ DSM 44095 [99.4%, 5] appear to members of the genus. Members of the species and genus are rare in nature, at least based on the habitats screened thus far as 16S rRNA in environmental samples and metagenomic surveys do not exceed 88-91% sequence similarity to the 16S rRNA gene sequence of strain NI 9100^T^ (U48996, X70425, X89947; status August 2009).

Figure 1a and Figure 1b show the phylogenetic neighborhood of *S. roseum* NI 9100^T^ in a 16S rRNA based tree. The sequence of the six 16S rRNA gene copies in the genome do not differ from each other, and are identical to the previously published sequence generated from DSM 43021 (X89947), whereas the sequence generated in the same year from the JCM 3005 version of strain 9100^T^ (U48996) differs by 24 nucleotides (1.7%).

**Figure 1a:**
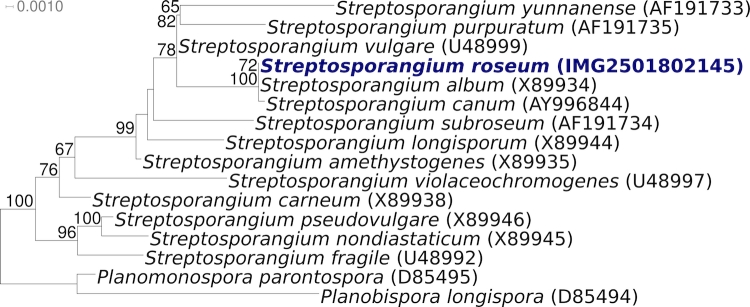
Phylogenetic tree highlighting the position of *S. roseum* NI 9100^T^ relative to the type strains of the other species within the genus (1a) except for *S. claviforme* (see text). The tree was inferred from 1,411 and aligned characters [8,9] of the 16S rRNA gene sequence under the maximum likelihood criterion [10] and either rooted with the results of Figure 1b (Figure 1a) or rooted in accordance with the current taxonomy. The branches are scaled in terms of the expected number of substitutions per site. Numbers above branches are support values from 1,000 bootstrap replicates if larger than 60%. Lineages with type strain genome sequencing projects registered in GOLD [11] are shown in blue, published genomes in bold.

**Figure 1b:**
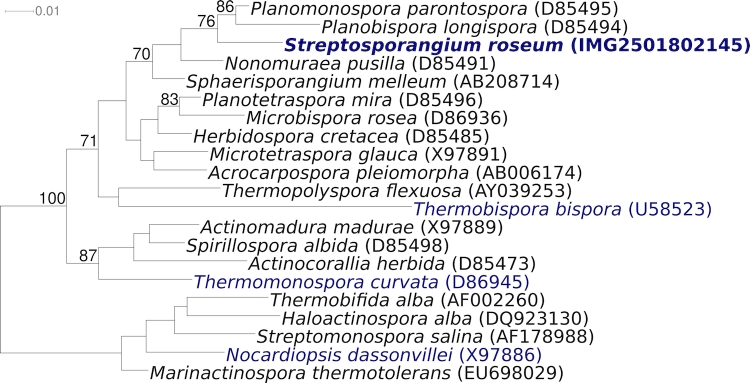
Phylogenetic tree highlighting the position of *S. roseum* NI 9100^T^ relative to the type strains of the other genera within the family *Streptosporanginea* . The tree was inferred from 1,369 aligned characters [8,9] of the 16S rRNA gene sequence under the maximum likelihood criterion [10] and either rooted with the results of Figure 1b (Figure 1a) or rooted in accordance with the current taxonomy. The branches are scaled in terms of the expected number of substitutions per site. Numbers above branches are support values from 1,000 bootstrap replicates if larger than 60%. Lineages with type strain genome sequencing projects registered in GOLD [11] are shown in blue, published genomes in bold.

**Figure 2:**
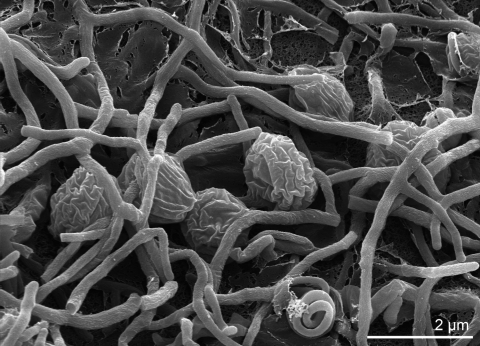
Scanning electron micrograph of *S. roseum* NI 9100^T^

A summary of the classification and features for *S. roseum* is listed in Table 1. We draw attention to the reader that we find quite an amount of contradictive results between old and more recent literature (see below). A potential but not ultimate source for this observation could be the usage of different experimental methods. A variety of media were used in the original description pertaining to cellular and mycelium morphology on (Figure 2) The color of the substrate mycelium is red-brown to yellow-brown [2,24]. Strain NI 9100^T^ utilizes glucose, arabinose, sucrose, xylose, fructose, and raffinose, but not inositol, mannose, rhamnose, or cellulose [19,20]. The strain is positive for arginine dihydrolase and acetoin production (Voges Proskauer test), weakly positive for citrate utilization, lysine decarboxylase, and ornithine decarboxylase, and negative for Kohn's gelatin gelatinase, urease, o-nitro-phenyl-galactoside β-galactosidase, tryptophan desaminase, tryptophan indole production, H2S production from sodium thiosulfate [19,20]. Starch hydrolysis and nitrate reduction are positive, but growth at 42°C and iodinin production are negative [24]. Mertz and Yao [18] reported that strain NI 9100^T^ can utilize glycerol, arabinose, rhamnose and inositol, which is in part contradictory to other results [20,21]. Gelatin is liquefied, milk is peptonized and red-brown to purple-brown soluble pigments are produced [18]. Zhang et al. [21] describe strain NI 9100^T^ as utilizing sorbitol and sorbose but to be negative for L-arabinose, erythrose, D-fructose, D-galactose, inositol, D-mannose, maltose, raffinose, and rhamnose, which again is in part in conflict with other studies [18-20]. Strain NI 9100^T^ produces a secondary metabolite, the antibiotic angucycline WS 79089B, which is an inhibitor of the endothelin-converting enzyme [20]. In contrast to *S. carneum*, strain NI 9100^T^ does not produce an antibiotic against *Staphylococcus aureus* [18].

**Table 1:** Classification and general features of *S. roseum* NI 9100^T^ according to the MIGS recommendations [12]

**MIGS ID**	**Property**	**Term**	**Evidence code**
	Current classification	Domain *Bacteria*	TAS [13]
		Phylum *Actinobacteria*	TAS [14]
		Class *Actinobacteria*	TAS [15]
		Subclass *Actinobacteridae*	TAS [15]
		Order *Actinomycetales*	TAS [15]
		Suborder *Streptosporangineae*	TAS [15]
		Family *Streptosporangiaceae*	TAS [16,17]
		Genus *Streptosporangium*	TAS [1-4]
		Species *Streptosporangium roseum*	TAS [1-4]
		Type strain NI 9100	
	Gram stain	not tested, probably positive	NAS [15,16]
	Cell shape	produces aerial mycelium	TAS [2]
	Motility	non-motile	TAS [2]
	Sporulation	non-motile spores	TAS [2]
	Temperature range	mesophile, temperature range not determined, does not grow at 42°C	TAS [1,18]
	Optimum temperature	28°C	TAS [1,18]
	Salinity	2.5% NaCl	TAS [19,20]
MIGS-22	Oxygen requirement	aerobic	TAS [2]
	Carbon source	several (see text), but be aware of contradicting results	TAS [19-21]
	Energy source	carbohydrates	TAS [19-21]
MIGS-6	Habitat	soil	TAS [2]
MIGS-15	Biotic relationship	free living	TAS [2]
MIGS-14	Pathogenicity	non pathogenic	NAS
	Biosafety level	1	TAS [22]
	Isolation	vegetable garden soil	TAS [2]
MIGS-4	Geographic location	most probably Chapel Hill, North Carolina, USA	TAS [2]
MIGS-5	Sample collection time	1955 or before	TAS [2]
MIGS-4.1 MIGS-4.2	Latitude, Longitude	35.913, -79.055	
MIGS-4.3	Depth	not reported	
MIGS-4.4	Altitude	not reported	

Evidence codes - IDA: Inferred from Direct Assay (first time in publication); TAS: Traceable Author Statement (i.e., a direct report exists in the literature); NAS: Non-traceable Author Statement (i.e., not directly observed for the living, isolated sample, but based on a generally accepted property for the species, or anecdotal evidence). These evidence codes are from of the Gene Ontology project [23]. If the evidence code is IDA, then the property was directly observed for a live isolate by one of the authors or an expert or mentioned in the acknowledgements.

The characteristics of the ribosomal protein AT-L30 of strain *S. roseum* JCM2178^T^ in comparison to other bacteria of the genus *Streptosporangium* is described elsewhere [25]. These data should be taken cautiously, as according to the Japanese Collection of Microorganisms (JCM) catalogue the strain number “JCM2178” is affiliated with *Aspergillus oryzae* (accessed to JCM in August 09), hence the true nature of strain *S. roseum* JCM2178^T^ in the study of Ochi [25] is unclear.

### Chemotaxonomy

The major fatty acids (relative ratio %) are iso-C_16:0_ (40.0), C_17:0 10-methyl_ (23.0), C_16:0_ (1.95), C_16:0_ _10-methyl_ (6.0), iso-C_14:0_ (14.0) (Reiner Kroppenstedt, personal communication). Partly different fatty acid patterns are reported elsewhere [18-20,26,27]. The proportions of diaminopimelic acid (A2pm) in the cell wall of strain *S. roseum* NI 9100^T^ are 71% meso-A2pm and 29% LL-A2pm [26]. The phospholipids of strain *S. roseum* NI 9100^T^ are phosphatidylethanolamine, hydroxyphosphatidylethanolamine, ninhydrin-positive and sugar-positive phospholipids, disphosphatidylglycerol, and posphatidylinositol [1]. The menaquinone compositions are MK-9 (III, VIII-H_4_) (56.5%), MK-9 (H_2_) (37.8%), MK-9 (H_0_) (5.0%), and MK-9 (H_6_) (0.7%) [1]. Galactose and madurose are present in whole cell sugars extracts, rhamnose is absent [1]. In general, the genus *Streptosporangium* is characterized by the whole-cell sugar type B or C, the phospholipid type IV and of the fatty acid type 3c [1].

## Genome sequencing and annotation

### Genome project history

This organism was selected for sequencing on the basis of its phylogenetic position, and is part of the *** G****enomic **** E****ncyclopedia of **** B****acteria and **** A****rchaea * project. The genome project is deposited in the Genome OnLine Database [11] and the complete genome sequence is deposited in GenBank. Sequencing, finishing and annotation were performed by the DOE Joint Genome Institute (JGI). A summary of the project information is shown in Table 2.

**Table 2:** Genome sequencing project information

**MIGS ID**	**Property**	**Term**
MIGS-31	Finishing quality	Finished
MIGS-28	Libraries used	Two Sanger libraries: 6kb pMCL200 and fosmid pcc1Fos One 454 Pyrosequence standard library
MIGS-29	Sequencing platforms	ABI3730, 454 GS FLX
MIGS-31.2	Sequencing coverage	8.45× Sanger; 27.6× Pyrosequence
MIGS-30	Assemblers	Newbler, phrap
MIGS-32	Gene calling method	Prodigal, GenePrimp
	INSDC ID	CP001814 (genome), CP001815 (plasmid)
	Genbank Date of Release	12/10/2009
	GOLD ID	Gc01156
	NCBI project ID	21083
	Database: IMG-GEBA	2501799901
MIGS-13	Source material identifier	DSM 43021
	Project relevance	Tree of Life, GEBA

### Growth conditions and DNA isolation

*S. roseum* NI 9100^T^, DSM 43021, was grown in DSMZ medium 535, Trypticase Soy Broth [28], at 28°C. DNA was isolated from 0.5-1 g of cell paste using the JGI CTAP procedure with modification ALM as described in [29].

### Genome sequencing and assembly

The genome was sequenced using a combination of Sanger and 454 sequencing platforms. All general aspects of library construction and sequencing performed at the JGI can be found at http://www.jgi.doe.gov/. 454 Pyrosequencing reads were assembled using the Newbler assembler version 1.1.02.15 (Roche). Large Newbler contigs were broken into 11,709 overlapping fragments of 1,000 bp and entered into assembly as pseudo-reads. The sequences were assigned quality scores based on Newbler consensus q-scores with modifications to account for overlap redundancy and to adjust inflated q-scores. A hybrid 454/Sanger assembly was made using the parallel phrap assembler (High Performance Software, LLC). Possible mis-assemblies were corrected with Dupfinisher [30] or transposon bombing of bridging clones (Epicentre Biotechnologies, Madison, WI). Gaps between contigs were closed by editing in Consed, custom primer walk or PCR amplification. A total of 2,837 Sanger finishing reads were produced to close gaps, to resolve repetitive regions, and to raise the quality of the finished sequence. The error rate of the completed genome sequence is less than 1 in 100,000. Together all sequence types provided 36.05× coverage of the genome. The final assembly contains 128,042 Sanger and 1,033,578 Pyrosequence reads.

### Genome annotation

Genes were identified using Prodigal [31] as part of the Oak Ridge National Laboratory genome annotation pipeline, followed by a round of manual curation using the JGI GenePRIMP pipeline (http://geneprimp.jgi-psf.org) [32]. The predicted CDSs were translated and used to search the National Center for Biotechnology Information (NCBI) nonredundant database, UniProt, TIGRFam, Pfam, PRIAM, KEGG, COG, and InterPro databases. Additional gene prediction analysis and functional annotation was performed within the Integrated Microbial Genomes - Expert Review (IMG-ER) platform [33].

## Genome properties

**Figure 3:**
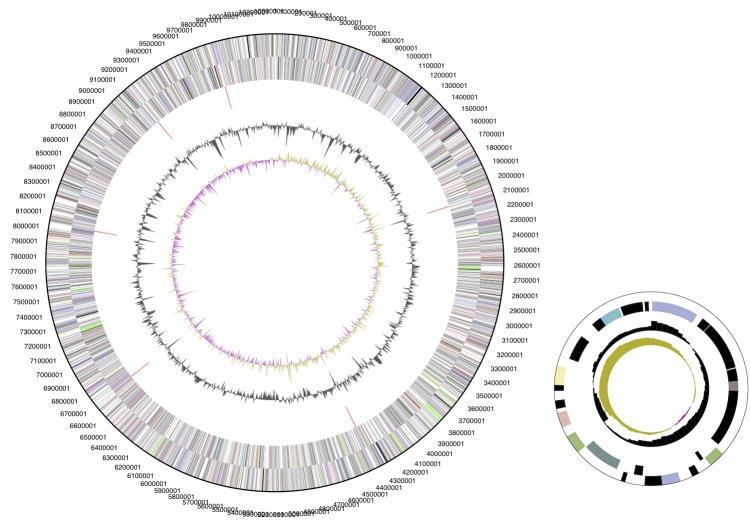
Graphical circular map of the genome; plasmid not to scale. From outside to the center: Genes on forward strand (color by COG categories), Genes on reverse strand (color by COG categories), RNA genes (tRNAs green, rRNAs red, other RNAs black), GC content, GC skew.

The genome consists of a 10,341,314 bp long chromosome and a small 28,204 bp plasmid with a 70.9% GC content (Table 3 and Figure 3). Of the 9,501 genes predicted, 9,421 were protein coding genes, and 80 RNAs. In addition, 446 pseudogenes were identified. The majority of protein-coding genes (62.5%) were assigned a putative function while those remaining were annotated as hypothetical proteins. The distribution of genes into COGs functional categories is presented in Table 4.

**Table 3:** Genome Statistics

**Attribute**	**Value**	**% of Total**
Genome size (bp)	10,369,518	100.00%
DNA coding region (bp)	9,121,910	87.97%
DNA G+C content (bp)	7,348,162	70.86%
Number of replicons	2	
Extrachromosomal elements	1	
Total genes	9,501	100.00%
RNA genes	80	0.84%
rRNA operons	6	
Protein-coding genes	9,421	99.16%
Pseudo genes	446	4.49%
Genes with function prediction	5,939	62.47%
Genes in paralog clusters	2,792	29.37%
Genes assigned to COGs	6,224	65.47%
Genes assigned Pfam domains	6,596	69.38%
Genes with signal peptides	2,248	23.65%
Genes with transmembrane helices	2,235	23.51%
CRISPR repeats	0	

**Table 4:** Number of genes associated with the general COG functional categories

**Code**	**value**	**%age**	**Description**
J	226	2.4	Translation, ribosomal structure and biogenesis
A	1	0.0	RNA processing and modification
K	966	10.3	Transcription
L	293	3.1	Replication, recombination and repair
B	1	0.0	Chromatin structure and dynamics
D	38	0.4	Cell cycle control, mitosis and meiosis
Y	0	0.0	Nuclear structure
V	189	2.0	Defense mechanisms
T	511	5.4	Signal transduction mechanisms
M	298	3.2	Cell wall/membrane biogenesis
N	2	0.0	Cell motility
Z	1	0.0	Cytoskeleton
W	0	0.0	Extracellular structures
U	43	0.5	Intracellular trafficking and secretion
O	167	1.8	Posttranslational modification, protein turnover, chaperones
C	424	4.5	Energy production and conversion
G	639	6.8	Carbohydrate transport and metabolism
E	600	6.4	Amino acid transport and metabolism
F	124	1.3	Nucleotide transport and metabolism
H	254	2.7	Coenzyme transport and metabolism
I	306	3.2	Lipid transport and metabolism
P	320	3.4	Inorganic ion transport and metabolism
Q	315	3.3	Secondary metabolites biosynthesis, transport and catabolism
R	974	10.3	General function prediction only
S	473	5.0	Function unknown
-	3187	33.8	Not in COGs
